# Mid-infrared integrated electro-optic modulators: a review

**DOI:** 10.1515/nanoph-2023-0286

**Published:** 2023-09-25

**Authors:** Tianqi Xu, Yuan Dong, Qize Zhong, Shaonan Zheng, Yang Qiu, Xingyan Zhao, Lianxi Jia, ChengKuo Lee, Ting Hu

**Affiliations:** School of Microelectronics, Shanghai University, Shanghai 201800, China; Department of Electrical & Computer Engineering, National University of Singapore, Singapore 117583, Singapore; Shanghai Institute of Microsystem and Information Technology (SIMIT), Chinese Academy of Sciences (CAS), Shanghai 200050, China

**Keywords:** mid-infrared, integrated photonics, optical modulators

## Abstract

Integrated mid-infrared (MIR) photonics have various applications in optical fiber communication, spectral detection and identification, free-space communication, and light detection and ranging, etc. The MIR electro-optic (EO) modulator, which is one of the key components of MIR integrated photonic systems, has attracted a lot of research interests. In this paper, we review the reported integrated MIR EO modulators based on different modulation mechanisms and material platforms. The recent research progresses and challenges of MIR EO modulators are presented and discussed. The unique advantages and the corresponding applications of each type of MIR modulators are summarized as well. In the end, we provide our perspectives of a few areas in integrated MIR modulators that are worthy for research attention in future.

## 1 Introduction

The wide spectrum of mid-infrared (MIR) wavelengths (2–20 µm) prompts a variety of exciting applications, including: (1) *Optical fiber communication*. As the demand for capacity in fiber-based telecommunication systems has shown dramatic growth in recent years, we are getting closer to the theoretical capacity limit of single-mode fibers (SMFs) [1]. New modulation techniques or new bands of spectrum are desired to increase the bandwidth capacity to keep up with the growing demand for communication capacity. Extending the optical communication bandwidth to ∼2-μm spectrum offers a promising solution to achieve cost-effective and low-energy data transmission in optical networks [2]. The implementation of 2-µm optical communication systems relies on the development of low-loss hollow photonic bandgap fibers (HC-PBGFs), which exhibit a low-loss window within the 1900–2100 nm band. The theoretical minimum achievable loss is below 0.1 dB/km [3]. Notably, researchers have successfully demonstrated a loss as low as 2.5 dB/km at 1990 nm for a 1.15 km long hollow fiber [4]. Furthermore, sulfur-doped fiber amplifiers (TDFAs) possess an optical gain window spanning 1910–2020 nm, and can be used in repeater systems for fiber optical communication in this wavelength range [5]. In addition, optical interconnect technology operating in the 2-µm band is expected to be used for fibreless chip-to-chip communications. (2) *Spectral detection and identification*. A lot of molecules display distinctive vibrational absorption peaks in the MIR region, making it suitable for spectral sensing with high sensitivity and selectivity. Specific applications include trace gas detection for environmental pollution monitoring [6, 7], protein analysis in bio-medicine [8], non-invasive diagnostics in medicine [9], etc. In recent years, lab-on-chip sensors which could provide *in-situ* gas detection or label-free biosensing have attracted great interests in both academic and industrial fields [10–13]. (3) *Free-space optical communication (FSOC)* [14]. Benefiting from the atmospheric transparent windows of 3–5 µm and 8–12 µm, FSOC offers an alternative solution to meet the growing demand for short-distance, high-capacity information transmission. With the advantages of the immunity to electromagnetic interference and the eye-safe optical power, it can be used for high-speed information communication between buildings in urban areas, emergency backup, and post-disaster recovery facilities. (4) *Beam steering system*. Beam steering system using the chip-scale optical phased arrays (OPAs) that operate in the atmospheric transparent bands can be used for robust light detection and ranging (LiDAR) [15, 16] and thermal imaging [17].

Modulator is one of the key components in optical systems. The aforementioned MIR applications drive the development of high-performance MIR modulators. Given the cost-effectiveness and the compatibility with complementary metal-oxide semiconductor (CMOS) processes, silicon photonics is very promising to achieve highly compact, power-efficient on-chip systems. Silicon photonic near-infrared (NIR) modulators have been studied over the last decades and have shown convincing performance, thus they are being converted to commercial applications. Owing to the remarkable achievements of NIR modulators, the research on MIR modulators using silicon photonics technology has attracted growing interests in recent years.

According to different optical guiding mediums and the corresponding electro-optic (EO) modulation mechanisms, the reported MIR modulators can be mainly categorized as follows: (1) Si, germanium (Ge), and Ge alloy-based modulators exploiting plasma dispersion effect and quantum-confined stark effect (QCSE). High-speed Si electro-refractive modulators have been widely studied as one of the functional components for 2-μm communication band. Because the optical loss induced by carrier absorption becomes aggravated as the wavelength increases, growing research efforts have been devoted to developing electro-absorption modulators in the longer MIR band using Ge and silicon–germanium (SiGe) materials, as well as the germanium-tin (GeSn)/silicon-germanium-tin (SiGeSn) quantum well. (2) Ferroelectric oxides modulators utilizing Pockels effect. Some ferroelectric oxides (e.g., LiNbO_3_ and BaTiO_3_) are linear electro-optic (Pockels) materials that can be utilized for integrated MIR modulators. Compared to the plasma dispersion effect in Si and Ge, ferroelectric oxides provide ultra-fast and pure refractive index modulation over a wide optical spectrum without additional absorption losses. (3) Two-dimensional (2D) material-based modulators rely on Pauli-blocking, quantum confined Franz–Keldysh (QCFK) effect, and Burstein–Moss (BM) effect. 2D materials integrated with Si substrates also show aspiring prospects in the MIR spectrum. In addition to graphene waveguide-based modulators, graphene plasmonic modulators are an emerging field of research that attracts great interests, owing to the features of ultra-compact size and substantially reduced light propagation loss at longer wavelengths [18, 19]. Moreover, black phosphorus (BP) modulators have been reported in recent years. Along with BP photodetectors [20] and BP light-emitting source [21], it may pave a promising path for the development of fully integrated MIR PIC systems.

This review recaps the representative MIR modulators reported over the last decade. From Section 2 to Section 4, various EO modulators classified according to the material and mechanism as mentioned above are introduced sequentially. Developments in the design and fabrication processes of MIR modulators are described. Typical examples of state-of-the-art MIR modulators and their critical performance limitations are discussed. In Section 5, different types of MIR modulators for various application prospects based on their unique properties and advantages are summarized. Moreover, outlooks on the promising approaches to further improve the performance metrics of waveguide-based MIR modulators are provided.

## 2 Si and Ge MIR modulators

Given the compatibility with mature CMOS processes, Si modulators in the SOI platform are highly competitive, and their working wavelength can be extended from NIR band to MIR band up to 3.8 μm [22]. The modulation mechanism in Si modulators is based on the free-carrier plasma dispersion effect, which is to tune carrier concentration by the external electrical field. As concentration variations of free-carriers impact the value of the complex refractive index, the predominance of the modulation on the real or imaginary part leads to the classification of electro-refractive modulators and electro-absorption modulators. In the telecommunication band, a great number of Si modulators based on the electro-refractive effect have shown excellent device performance that supports complex modulation formats and long-distance information transmission [23]. In recent years, the operating wavelength of Si electro-refractive modulators has been extended to the 2-μm band, showing convincing performance [24–26]. However, in the MIR band larger than 3 μm, since the optical losses caused by carrier absorption increase with wavelength [27], it provides an alternative approach for the modulation of light intensity directly through the electro-absorption effect.

Additionally, since SiO_2_ cladding causes unacceptable absorption loss in the spectrum beyond 3.6 μm, Ge has been explored to bridge the gap of SOI for applications in the longer MIR band and to open up a wide space for future innovations in high-performance modulators. Ge electro-absorption modulators (EAMs) exploiting the plasma dispersion effect were reported to operate up to 8 μm [28], and the maximum operating wavelength of a silicon-germanium (SiGe) EAM was reported to be ∼11 μm [29]. In addition, other electro-absorption effects (i.e., quantum confinement Stark effect (QCSE) [30] and Franz–Keldysh (FK) effect [31]) in Ge-based heterogeneous structures were utilized to realize the absorption-based intensity modulation in the MIR range.

In this section, we mainly organize the content as MIR electro-refractive modulator and electro-absorption modulator using Si and Ge material platforms.

### 2.1 MIR electro-refractive Si and Ge modulators

**Figure 1:**
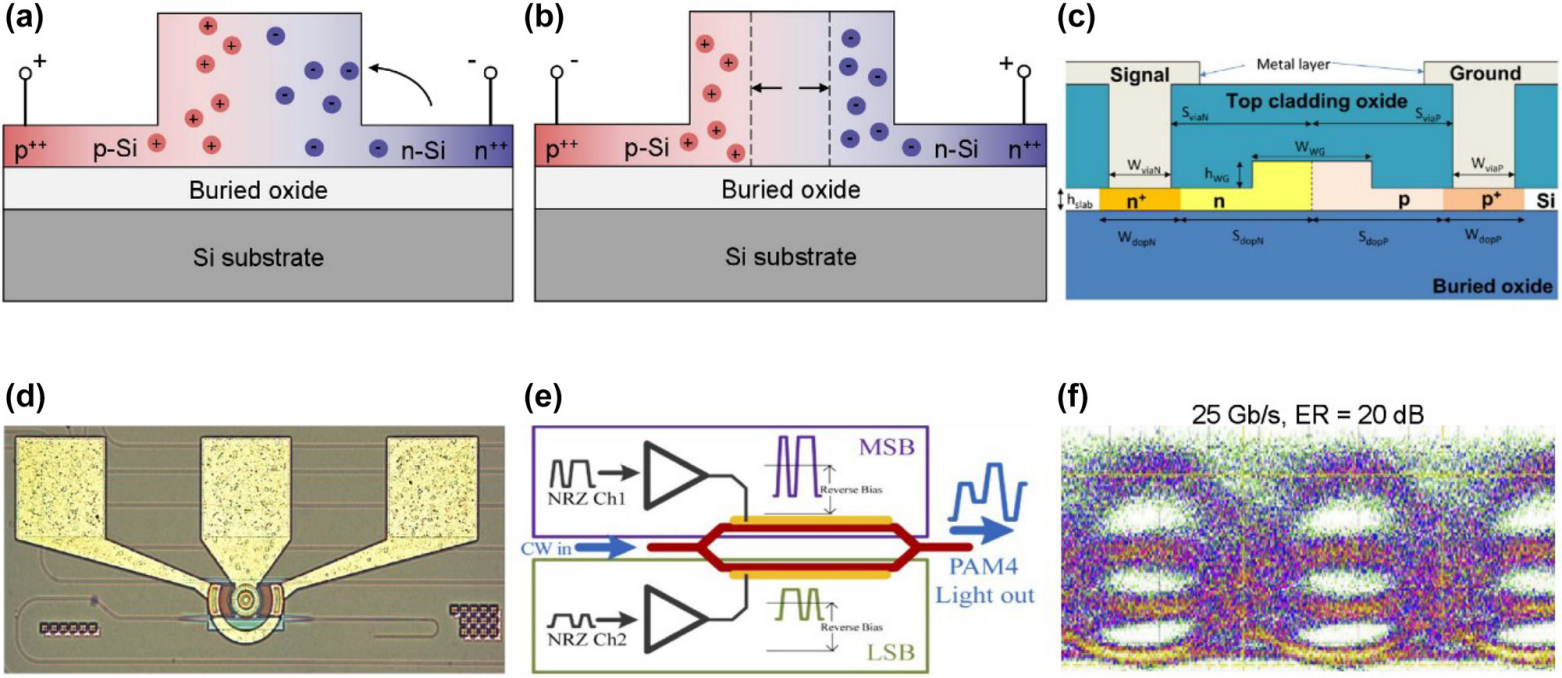
Representative MIR electro-refractive type Si modulators. (a) Cross-section of PN diode with carrier-injection state. (b) Cross-section of PN diode with carrier-depletion state. (c) Cross-section of PN diode of Si MZM at 2-μm wavelength. (d) Optical microscopy image of Si MRRM at 2-μm wavelength. (e) Schematic of the PAM-4 modulation format in the dual-drive MZM working at 2-μm wavelength. (f) Eye diagram of Si MZM with an ER of 20 dB and a 25 Gb/s data rate. Figures reproduced with permission from: (c), (d) Ref. [2], licensed under Creative Commons Attribution 4.0 License; (e), (f) Ref. [34], licensed under Creative Commons Attribution 4.0 License.

The operation mechanism of a Si or Ge electro-refractive modulator is based on the plasma dispersion effect. Among the plasma dispersion modulators that have been reported so far, there are three carrier manipulation approaches: carrier injection, carrier depletion, and carrier accumulation. The refractive index can be tuned by two types of electrical structures built in waveguides. Either in a carrier-injected or -depleted PN/PIN diode [Figure 1(a) and (b)] or a carrier-accumulated Si-insulator-Si (SIS) capacitor. According to the optical structures, the electro-refractive modulators can be divided as Mach–Zehnder modulators (MZMs) and micro-ring resonator modulators (MRRMs). When the plasma dispersion effect is exploited at the shorter MIR band (<4 μm), the electro-refractive induced phase shift of the light is dominant, while the change of light intensity is considered an unwanted side effect because it degrades the extinction ratio (ER) [27].

#### 2.1.1 MIR electro-refractive Si modulators

Since the discovery of low-loss hollow-core photonic bandgap fibers (HC-PBGFs) at 1.95 μm [32] as well as the optical gain window of thulium-doped fiber amplifiers (TDFAs) at around 2 μm [5], the 2-μm band has been considered as a new communication band that can achieve high data rates and provides a promising solution to the congestion problem of existing communication bands. Therefore, it is necessary to develop modulators operating around the 2-μm region, as it is one of the key optical components of communication systems. The design of Si electro-refractive modulators for the 2-μm band is directed towards high modulation rates (tens of Gbit/s), low power consumption (∼pJ/bit), and large ER to meet the needs of end-users for long-distance data transmission.

The MIR Si modulator working around 2 μm was first demonstrated in 2012 [33]. This carrier-injection modulator exhibits the 3 dB bandwidth of 1.8 GHz and bit rates of 3 Gbit/s. Prof. Mashanovich et al. reported both Si MZMs and MRRMs in 2018 [2]. Using the PN diode shown in Figure 1(c), the MZM exhibits the modulation efficiency (*V*_π_⋅*L*) of 2.68 V⋅cm under 4 V DC voltage and a 13 dB insertion loss. The eye diagram shows that the AC ER is 5.8 dB with a 20 Gbit/s data rate. Since there is a significant improvement in data rate as compared to Ref [33], this study paves the way for the commercialization of optical communications in the 2-μm band. Moreover, they presented an MRRM as shown in Figure 1(d) with low power consumption (2.38 pJ/bit) and an ER of 2.3 dB at a data rate of 3 Gbit/s. Later, the same group demonstrated another carrier-depletion MZM [34]. Using the streamlined PAM-4 modulation format illustrated in Figure 1(e), this dual-drive MZM shows significant improvements in insertion loss (4.96 dB), data rate (25 Gbit/s, as shown in Figure 1(f)), and ER (20 dB) compared to the previously reported Si modulators.

Silicon modulators applied to the 2-μm optical communication band have been widely investigated and the performance of the devices has been improved in recent years. However, it is noteworthy that the driven voltage of some reported Si MZMs is higher than the advanced CMOS ICs operation voltage. In order to reduce the driven voltage to about 2 V, introducing new modulation-enhanced structures, such as photonic crystal cavities into the MZI waveguide could be considered [35]. Additionally, an 80 Gbit/s data rate can be obtained at 2 μm either by using the 16-QAM filter bank multicarrier (FBMC) modulation format [36] or using the 4-level pulse amplitude modulation (PAM-4) format [26]. Therefore, to further increase the data rate on a single carrier wavelength, the architectures of the modulators need to be optimized to satisfy advanced modulation formats.

#### 2.1.2 MIR electro-refractive Ge modulators

Given the compatibility with mature CMOS processes, Ge is becoming an important candidate with great potential to extend the operating wavelength range of MIR modulators up to ∼11 µm [29]. On Ge-on-insulator (GOI) platform, MRRM with a 13 dB extension ratio operating at 2 µm was demonstrated [37]. On Ge-on-Si (GOS) platform, a thermo-optic modulator with 16 mW power consumption (for 2π phase shift) at 5 μm was reported [38].

**Figure 2:**
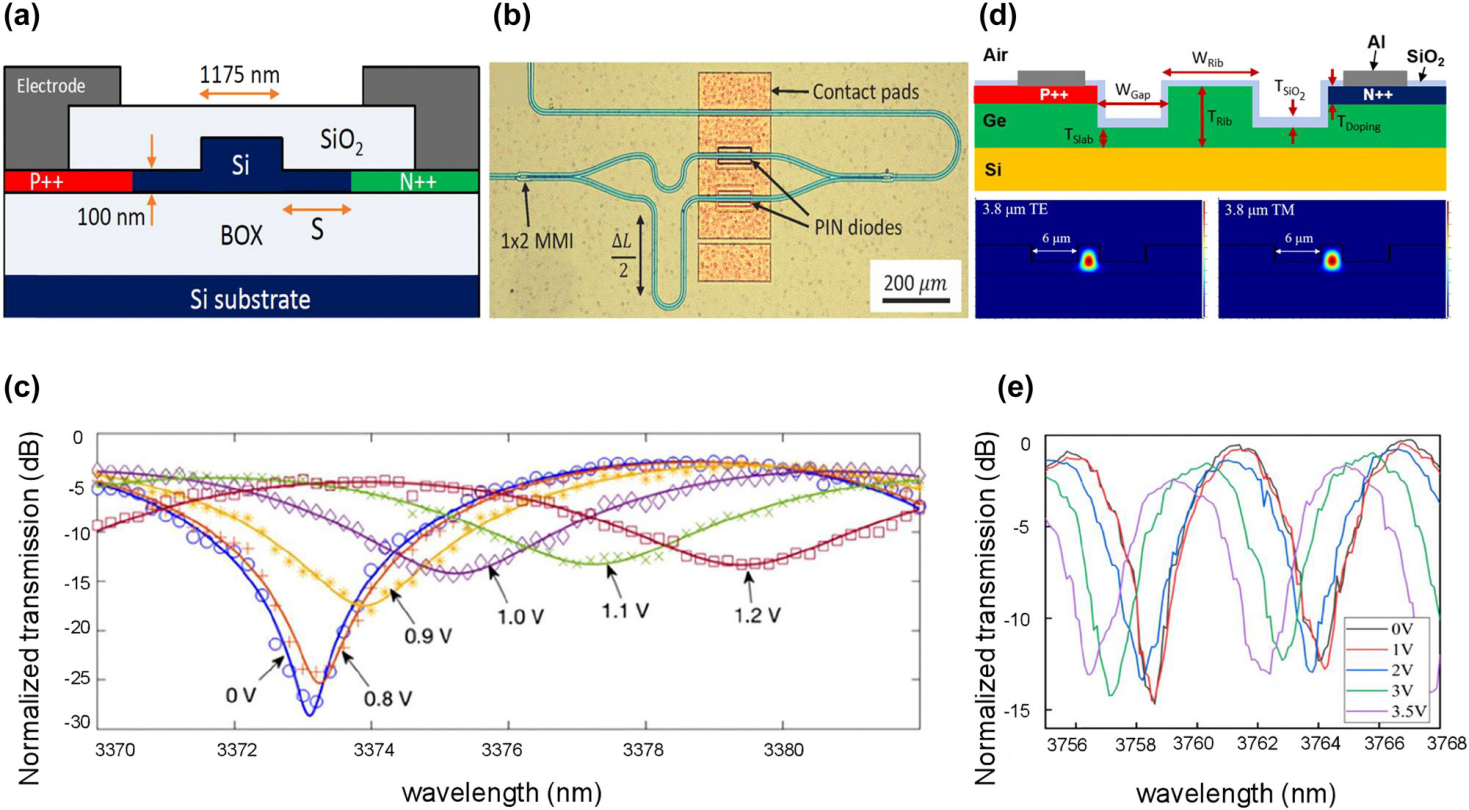
Representative MIR electro-refractive type Si and Ge modulator. (a) Cross-section of PIN diode of Si carrier-injection MZM at 3.8-μm wavelength. (b) Optical microscopy image of the Si MZM. (c) Normalized optical transmission versus wavelength under different DC voltages. (d) Cross-section of PIN diode of Ge carrier-injection MZM at 3.8-μm wavelength. (e) Optical transmission as the function of wavelength. Several applied DC voltages are shown in the inset. Figures reproduced with permission from (a), (b), (c) Ref. [22], licensed under Creative Commons Attribution 4.0 License. (d), (e) Ref. [28], licensed under Creative Commons Attribution 4.0 License.

As predicted by Prof. Mashanovich, free-carrier absorption is significantly larger in Ge than in Si and the situation becomes more severe at the longer wavelength range [39], Ge electro-refractive modulators usually have worse performance than their Si counterparts. Both SOI and GOS electro-refractive modulators operating at 3.8 µm were reported by Prof. Mashanovich’s research group [22, 28]. The cross-sections of the PIN diodes of Si and Ge MZM are shown in Figure 2(a) and (d), respectively, as well as their corresponding DC characterizations which are shown in Figure 2(c) and (e). The experimental results show that the Si MZM exhibits better performance than Ge MZM, in terms of modulation efficiency *V*_π_⋅*L* (0.0052 V⋅cm vs. 0.47 V⋅cm), ER (22.2 dB vs. 13 dB), and power consumption (26.5 pJ vs. 9900 pJ). The better modulation efficiency is caused by the change of the refractive index in Si is higher than that of Ge with the same concentration variation of free carriers. Thus, the relatively shorter modulation length *L* (100 µm) [Figure 2(b)] and the lower bias voltage is needed in the Si modulator to make the phase change of π. The comparatively poor ER and power consumption of the Ge MZM are due to the more significant carrier absorption in Ge (an injection current of 0.23 A produces a carrier absorption of 3.85 dB in Ge MZM). In an electro-refractive modulator, the free-carrier absorption is an unwanted side effect. It will make the electro-optical phase modulator suffer from a certain degree of parasitic amplitude modulation, which will not only lead to additional insertion loss but also affect the ER of the modulated signal when further constructing an MZI-type intensity modulator.

Compared with the GOS platform, the GOI platform seems to be a preferable choice for a shorter MIR band. Since the GOI platform provides better optical confinement, the waveguide has less propagation loss than the GOS platform. In Ref [37], based on numerical analysis, the phase modulation efficiency of the GOI modulator operating around the 2-µm band is about 10 times higher than that of the GOS modulator. The fabricated Ge ring resonator exhibits 13 dB modulation depth with a 1 mA injection current. Compared with MZMs on the GOI platform [40], a micro-ring resonator significantly reduces both device footprint and power consumption.

Moreover, according to Ref. [41], the turn-on voltage of the GOI modulator will be lower compared to the Si modulator due to the narrower bandgap of Ge. The power consumption of the GOI carrier-injection modulators could be lower than their Si counterparts. Therefore, Ge modulators still have a great potential for better performance by several methods mentioned below: (1) Use advanced processing techniques such as laser annealing, flash lamp annealing as well as the spin-on-glass method to achieve higher effective doping and improve junction quality [41]. (2) Improve film quality of Ge to minimize the impact on carrier lifetime *τ* due to crystal defects near the Ge/Si interface for higher modulation speed [40]. (3) Optimize the design of the electrodes for higher modulation speed and introduce lightly doped areas adjacent to the heavily doped regions to reduce the series diode resistance for larger electrical bandwidth [28]. Additionally, to our best knowledge, there have been no carrier-depletion Ge modulators reported that work in the MIR band. Owing to the smaller junction capacitance of the carrier-depletion modulator, it exhibits larger RC bandwidth, faster modulation speed, and lower power consumption.

Several novel electrical structures have been proposed in the NIR band to improve modulation efficiency, such as interleaved waveguide, where *P* and *N* doping areas alternate in the direction of waveguide propagation [42], L-shaped, U-shaped doping [43], and interdigitated PN junctions around the circumference of the MRRM [44]. These innovative structures, in combination with the inherently promising performance of carrier depletion modulators, may open up new opportunities for Ge MIR modulators.

### 2.2 MIR electro-absorption Si and Ge modulators

This section includes electro-absorption modulators (EAMs). The fundamental device configuration of an EAM is a single waveguide, where the optical intensity is directly tuned by the applied electrical fields. Besides the conventional Si or Ge ridge waveguides with PIN/PN diodes, graded-index SiGe waveguides with Schottky diodes, and Ge rib waveguides integrated with Ge/SiGe or GeSn/SiGeSn multiple quantum well (MQW) can also act as optical-intensity-modulating components.

#### 2.2.1 MIR electro-absorption Si modulators

Free carriers cause the absorption loss of light propagating in the waveguide, leading to the attenuation of the optical intensity. In longer wavelength ranges, especially beyond 4 μm, free carriers-induced losses are greater than those in NIR wavelength range, thus hindering the realization of phase modulation and phase modulation-based advanced modulation formats [27]. Therefore, Si modulators based on the plasma dispersion effect are more suitable to modulate the light intensity directly at longer MIR wavelengths. Although a single waveguide Si EAM could be considered to be an intensity modulator, the high absorption of the cladding layer (SiO_2_) in the 2.6–2.9 μm range and beyond 3.6 μm limits the wider utilization of SOI platforms [45].

#### 2.2.2 MIR electro-absorption Ge modulators

MIR modulators are key components for applications in free-space communications and chip-scale spectral sensors, however, Si MIR modulators working in the 4–12 µm band have rarely been reported because of the limitations of the SOI platform in the >4-µm band.

Ge has a wide transparency window from 1.8 to 14 µm and the absorption coefficient of Ge increases with the wavelength. Moreover, as predicted in Ref. [39], the electro-absorption effect of Ge is more significant than Si at the same wavelength. This indicates that in the MIR range beyond 4 µm, Ge EAM is more efficient than those realized by Si EAM. This theory has been experimentally proved by the Ge-rich index-graded SiGe waveguides in Ref. [46], where one can observe that the modulation depth increases with the wavelength varying from 5 to 9.5 µm.

**Figure 3:**
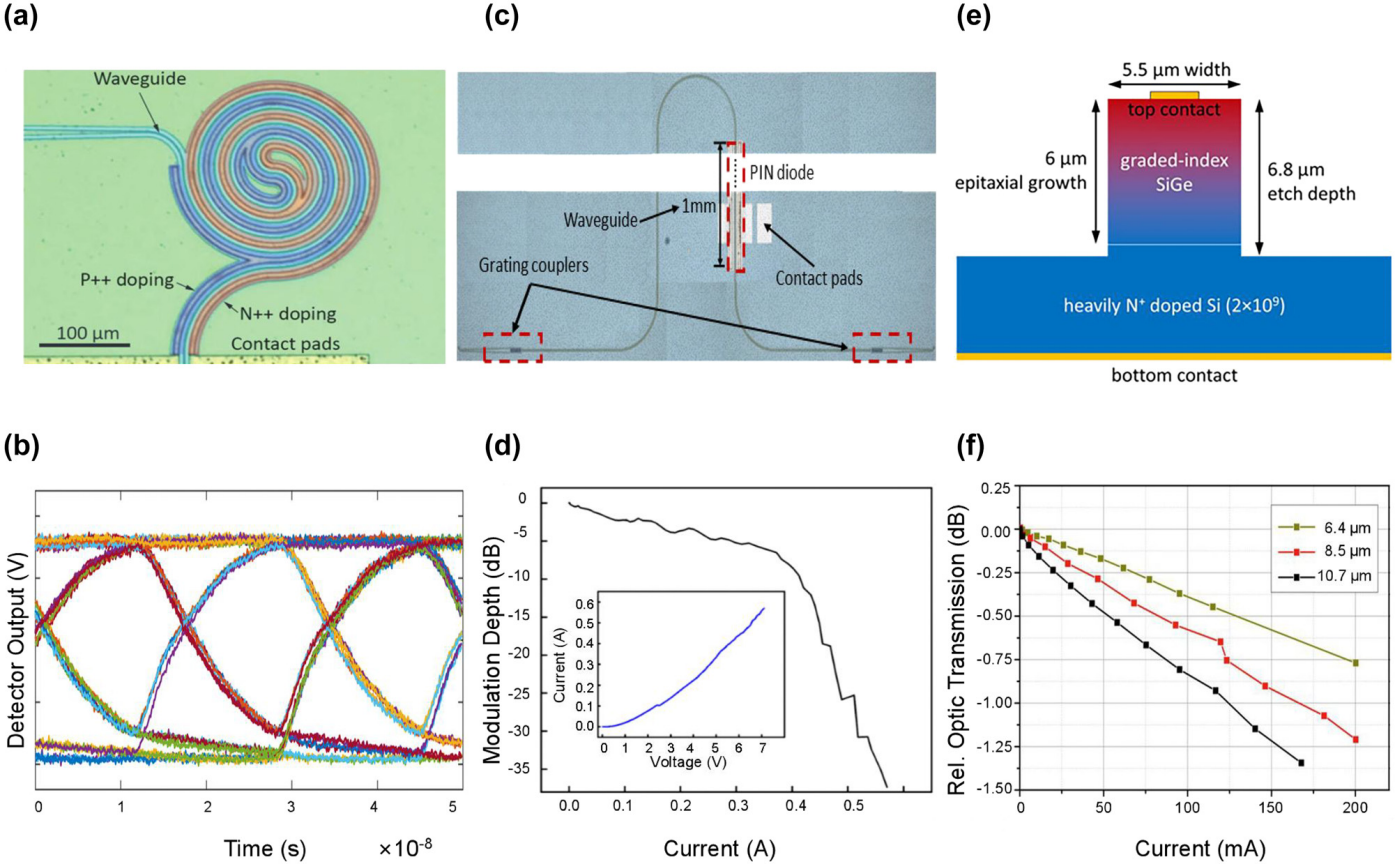
Representative MIR electro-absorption type Si and Ge modulators. (a) Optical microscopy image of the spiral-shaped Si EAM at 3.8-μm wavelength. (b) Eye diagram of Si EAM at 60 Mb/s data rate. (c) Optical microscopy image of the single-waveguide Ge EAM at 3.8-μm wavelength. (d) Modulation depth versus current of the Ge EAM. (e) Cross-section of the schematic of the SiGe EAM. (f) Static optic transmission as a function of the current of the carrier-injection EAMs operating at several wavelengths. Figures reproduced with permission from: (a), (b) Ref. [22], licensed under Creative Commons Attribution 4.0 License; (c), (d) Ref. [28], licensed under Creative Commons Attribution 4.0 License; (e), (f) Ref. [29], licensed under Creative Commons Attribution 4.0 International License.

Both SOI and GOS electro-absorption modulators (EAMs) operating at 3.8 µm were reported by Prof. Mashanovich et al. [22, 28]. Compared with spiral-shaped Si EAM [Figure 3(a)], which has a DC modulation depth of 34 dB under 2 V driven voltage and a data rate of 60 Mbit/s [Figure 3(b)], the Ge EAM in Figure 3(c) shows a comparable modulation depth of 35 dB, but with a driven voltage of up to 7 V [Figure 3(d)]. This is caused by the weaker confinement of light on the GOS platform, which results in a larger dimension of Ge waveguides than Si waveguides and a low concentration of free carriers in waveguides under the same driven voltage. Therefore, a higher driven voltage is required to achieve a comparable modulation depth.

The maximum working wavelength of Ge EAM exploiting plasma dispersion effect is currently at 8 µm, which exhibits 2.5 dB ER with 7 V DC forward bias in a 2-mm-long PIN diode [28]. In addition to Ge, silicon–germanium (SiGe) alloys have shown promising potential in MIR photonics. Miguel Montesinos-Ballester et al. first experimentally demonstrated an all-optical SiGe modulator operating at 11-µm wavelength [46]. Recently, the same group reported SiGe EAMs exploiting the free-carrier plasma effect with the operating wavelengths of 6.4, 8.5, and 10.7 μm, respectively [29]. For better light confinement in longer MIR bands, the graded refractive index profile of SiGe waveguides is used, which is shown in Figure 3(e). Different from the PIN structure in conventional plasma dispersion effect-based modulators, the manipulation of carrier concentration in graded-index SiGe modulators is realized by the Schottky diode. Additionally, since the refractive index in the SiGe waveguide increases linearly with the composition of Ge, the optical modes are confined to the top of the waveguide, so that it is away from the highly N-doped Si substrate, which avoids excess optical losses. As shown in Figure 3(f), the maximum modulation depth is 1.3 dB at 10.7 μm with an injection current of 170 mA. To achieve a higher modulation speed, a modulator working in the depletion regime has also been proposed, showing an electrical bandwidth of 225 MHz. Subsequently, within the same year, the research group performed additional design optimizations on the structure of grounded coplanar waveguide (CPWG) electrodes to achieve a 50-ohm impedance match and mitigate the velocity mismatch between the RF signal and the optical waves transmitted through the waveguide. Consequently, the modulation bandwidth of the graded SiGe modulator was further enhanced to 1 GHz [47]. In conclusion, Ge modulators working in the >4-µm band still have considerable prospects for research to improve the device performance including modulation depth, electrical bandwidth, etc. for practical applications in the long MIR band. For example, the increase of modulation depth could improve the signal-to-noise ratio (SNR) of the MIR spectroscopy.

Ge/SiGe quantum well stacks can be epitaxially grown on top of the Ge-buffer layer on Si substrates, provided the presence of separate-confinement heterostructures (SCH) layer between the well-layer and barrier-layer. The SiGe material system is compatible with standard Si fabrication processes. A simulation study reported in Ref. [48] demonstrated the intensity modulation at wavelengths of 6.6 µm, 7.3 µm, and 10 µm through the modeling and analysis of two different asymmetric quantum well structures i.e., the step quantum well (StQW) and the triangular quantum well (TQW). The results show an ER up to 1 dB and a modulation speed reaching several tens of GHz in a 100-µm device. Additionally, unlike Ge, which has an indirect bandgap, the GeSn alloys exhibits reduced direct bandgap by incorporating varying amount of Sn to Ge, which broadens the operating wavelength range and increases the intrinsic absorption and radiation coefficients of light [31]. The strong electro-absorption based on the QCSE in the GeSn/SiGeSn multiple quantum well (MQW) was demonstrated around 2 μm [30]. This MQW structured EAM exhibits a 6.87 dB ER and 27 GHz 3-dB bandwidth with 215-μm modulation length. Compare to a 250-μm-long carrier-injection Ge variable optical attenuator (VOA) working in the same wavelength range [40], the MQW structured EAM shows 9 times 3-dB bandwidth of the Ge VOA, making it promising for applications in 2-μm optical communication systems.

### 2.3 Benchmark of Si and Ge MIR modulators

Table 1 summarizes MIR modulators exploiting the plasma dispersion effect and QCSE reported in recent years, covering the modulation scheme and several critical performance parameters including modulation efficiency, 3-dB electrical bandwidth, ER, insertion loss, and power consumption.

**Table 1:** Benchmark of Si and Ge MIR modulators.

Year	Wavelength (μm)	Material platform	Structure	*V*_π_·*L* (V⋅cm)	ER (dB)	Speed/3 dB bandwidth	Insertion loss (dB)	Switching voltage/current	Switching energy/power	Ref.
2021	1.95	SOI	MZM Carrier-depletion	1.6	22 DC	18 GHz 80 Gbit/s	15	2 V	NA.	[26]
2018	1.95	SOI	MZM Carrier-depletion	2.68	5.8 AC	75 GHz 20 Gbit/s	13	4 V	NA.	[2]
			Ring Hybrid carrier-injection and -depletion	4.4−6.4	2.3 AC	3 Gbit/s	NA.	1–10 V	2.38 pJ/bit	
2020	1.95	GOI	EAM Carrier-injection	NA.	7 DC	30 MHz	NA.	2.3 V	NA.	[40]
2020	2	SOI	MZM Carrier-depletion	2.89	10.3 AC	25 Gbit/s	4.96	4 V	NA.	[25]
2021	2	GOI	Ring Carrier-injection	NA.	13 DC	NA.	NA.	1 mA	NA.	[37]
2021	2	SOI	Ring Carrier-depletion	0.975	>15 DC	15 GHz 45 Gbit/s	∼8	2 V	NA.	[24]
2022	2	SOI	Ring Carrier-depletion	0.85	>15 DC	18 GHz 50 Gbit/s	8	4 V	NA.	[49]
2018	2	GeSn/SiGeSn MQW-on-GOS	EAM QCSE	NA.	6.87 DC	27 GHz	3.97	4.2 V	NA.	[30]
2012	2.165	SOI	MZM Carrier-injection	0.012	23 DC	1.8 GHz	9.0	0.91 V	NA.	[33]
2019	3.8	SOI	EAM Carrier-injection	NA.	34 DC	60 Mbit/s	2.9	2 V 0.089 A	2970 pJ	[22]
			MZM Carrier-injection	0.0052	22.2 DC	125 Mbit/s	∼1	1.2 V	26.5 pJ	
2019	3.8	GOS	EAM Carrier-injection	NA.	>35 DC	60 MHz	NA.	7 V 0.575 A	67 nJ	[28]
			MZM Carrier-injection	0.47	13 DC	NA.	3.5 V 0.17 A	9.9 nJ 595 mW	
	8		EAM Carrier-injection	NA.	2.5 DC	NA.	NA.	7 V 1.04 A	7.28 W	
2022	10.7	Graded-index SiGe	EAM Carrier-depletion	NA.	1.3 DC	225 MHz	15.6	4 V	NA.	[29]
2022	5.5–9	Graded-index SiGe	EAM Carrier-depletion	NA.	1 DC	1 GHz	2.5 @5.5 µm 10.5 @9 µm	4 V	NA.	[47]
2022	6.6–10	Ge/SiGe QWs	EAM QCSE	NA.	1 DC	60 GHz	2.5 @7.3 µm 1.5 @10 µm	12 V	NA.	[48]

Si modulators using the electro-refractive effect show advantages in the shorter MIR band (<4 µm). As seen in Table 1, Si MZMs operating in the depletion regime provide a reliable solution for extending the current telecom window to the 2-µm band with their large frequency response up to 75 GHz [2], a high data rate of 80 Gbit/s [26], as well as the small power consumption of 2.38 pJ/bit [2]. Furthermore, a carrier-depletion Si MRRM was reported in 2022 [49], which shows significant performance improvements, in terms of ∼7.5 times modulation efficiency (0.85 V⋅cm vs. 6.4 V⋅cm) and ∼17 times modulation speed (3 Gbit/s vs. 50 Gbit/s) as compared to a hybrid carrier-injection and -depletion MRRM working in the same 2-µm band [2]. Currently, the maximum operating wavelength of the carrier-injection-based Si MZM is 3.8 µm [22]. Although this modulator shows a modulation efficiency of 0.052 V mm with a 100-µm modulation length, it has a limited modulation speed of 125 Mbit/s.

In the longer MIR band (>4 µm), research is still in the infancy stage. The reported modulators listed in this table are all electro-absorption types. GOS, as well as SiGe alloys on Si substrates, are two promising material platforms for MIR EAMs which are feasible for applications such as on-chip choppers and optical switches [50]. Since the refractive index contrast of the GOS platform is not as large as that of the GOI platform, the waveguides are broadened. This increases the Ohmic contact separation and requires a larger drive voltage. Therefore, the compact and power-efficient EAM is the objective for further research.

## 3 Ferroelectric oxides MIR modulators

**Figure 4:**
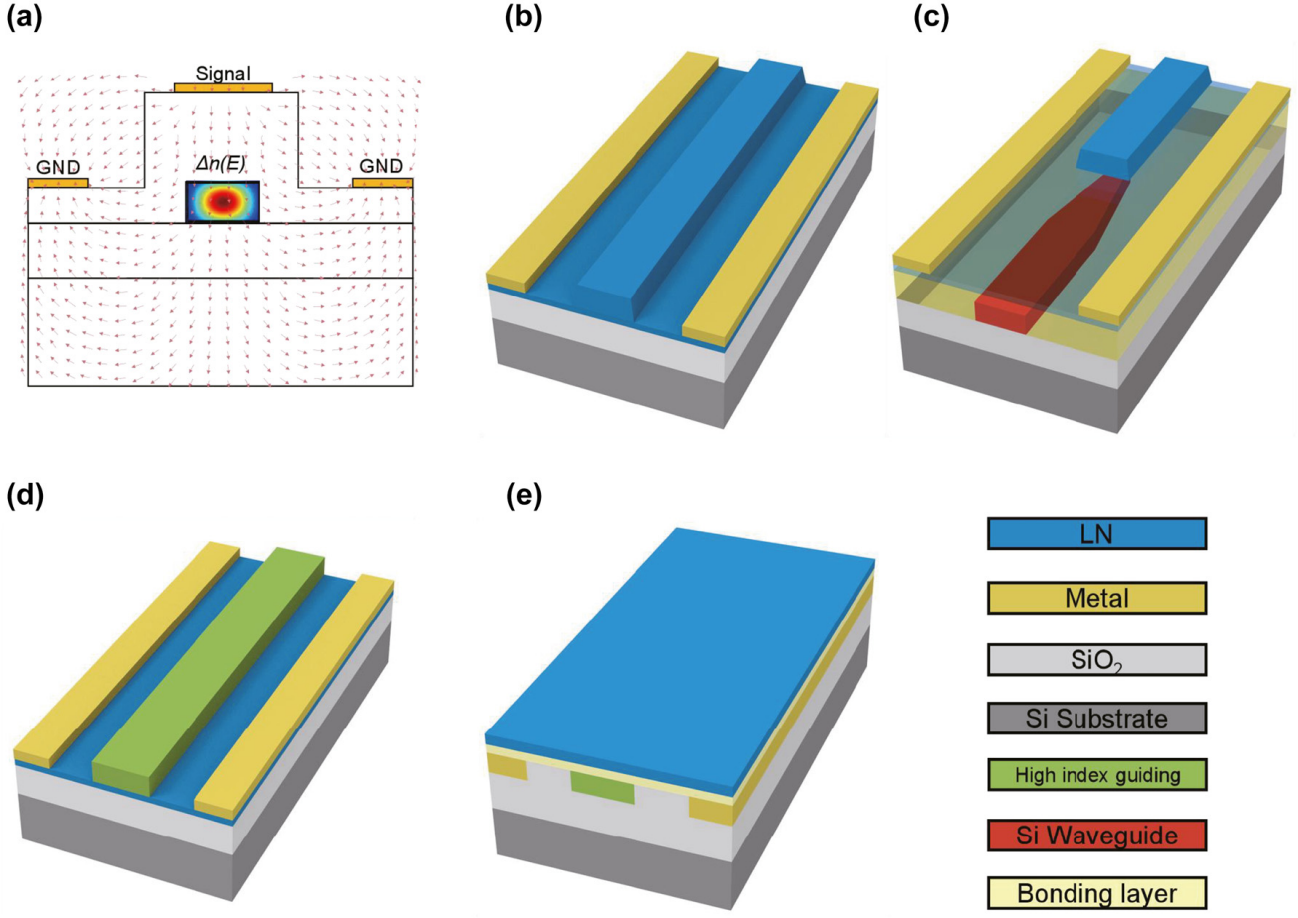
The electric field distribution and the device configurations of MIR LN modulators. (a) Cross-section of *z*-cut MIR LN modulators with applied electric field. Design of *x*-cut MIR LN modulators using (b) an LN ridge waveguide, (c) a hybrid LN waveguide and Si waveguide, (d) rib-loaded LN thin film, and (e) a buried waveguide with LN thin film.

Ferroelectrics are materials with non-centrosymmetric crystal structures. According to the characteristics of structural changes during phase transition from paraelectric to ferroelectric, they can be classified into water-soluble ferroelectrics (e.g., Potassium dihydrogen phosphate (KDP), and admixturedtriglycinesulphate (TGS)) and ferroelectric oxides (e.g., lithium niobate, (LN) and barium titanate (BTO)). In such ferroelectric materials with large dielectric constants and spontaneous polarization, a large Pockels effect (linear electro-optic effect) can be observed. In contrast to plasma dispersion-based modulators, in which the involved current flow results in EO response limited either by the carrier lifetime under forward bias or by the RC characteristics under reverse bias, Pockels modulators show ideal EO modulation with picoseconds-scale modulation speed and low power consumption. As shown in Figure 4(a), the applied electric field modifies the optical properties (refractive index, dielectric constant, birefringence, etc.) of the crystal. Consequently, changes in the orientation and length of the principal axis of the index ellipsoid induce changes in the eigenmodes and eigenvalues of the transmitted beam, which correspond to modulation of the polarization and phase of the light [51].

In this section, two Pockels effect-based modulators are introduced, which are lithium niobate (LN) modulators and barium titanate (BTO) modulators. The fabrication processes of both Pockels thin films and the structure of the phase shifters are introduced sequentially. Moreover, the importance of crystalline anisotropy on the performance of Pockels modulators is underlined. At last, several high-performance MIR Pockels modulators are compared and evaluated, and an outlook is given.

### 3.1 LN MIR modulators

LN has an EO coefficient of approximately 30 pm/V. In the last decades, the integrated LN modulators have been widely investigated and shown convincing performance in the NIR band. Therefore, the motivation of the paradigm shift from commercial bulk LN modulators to on-chip LN modulators has catalyzed great breakthroughs in both fabrications of lithium niobate-on-insulator (LNOI) wafers and the etching processes of LN thin films [52, 53]. Monocrystalline LNOI wafers are commercially available using the “Smart-cut” ion slicing method [52]. Currently, the commercially available wafer size of LNOI wafers in the current market is 4 inches only [54]. They are much more expensive than SOI wafers and are not compatible with 8-inch or 12-inch Si photonics fabrication lines [55]. The characteristics of the LN material led to the etching process being the core technical challenge for the entire LN integrated optics. LN waveguides with smooth sidewall (e.g., 0.452 nm [56]) and low optical loss (e.g., 0.027 dB/cm [53] at the wavelength of 1560.48 nm) can be achieved through variable etching methods including photolithography-assisted chemo-mechanical etching [57], gas clustered ion beam smoothening [58], and Ar^+^ plasma etching [59].

Integrated LN modulators can be divided into two main categories according to whether the LN films are etched or not. Etched LN waveguide-based modulators become the preferred scheme benefiting from their decreased low propagation loss and high electro-optical overlap. One method is to deposit electrode material directly on both sides of an *x*-cut LN ridge waveguide, the schematic is shown in Figure 4(b). Another method uses the direct bonding method to couple conducted light upwards from the Si waveguide into the LN waveguide via vertical adiabatic couplers (VACs) for optical phase modulation, as illustrated in Figure 4(c) [60]. This approach allows the integration of LN components with other Si components in the same substrate.

Some designs avoid direct patterning on LN, and the LN thin films are integrated with high-refractive-index guiding materials to circumvent the complex etching processes of LN. However, such schemes come at the expense of modulation efficiency as the light mode is partially confined in the LN thin films [60–62]. According to the relative position of the LN thin film and the waveguide, there are two different types. One is the rib-loaded structure where waveguide material such as TiO_2_ [63] and a-Si [64] are deposited on top of the LNOI platform (geometry shown in Figure 4(d)), another is bonded structure, where the active layer LN is covered on top of the SOI platform [Figure 4(e)]. The main purpose of the second scheme is to achieve low-loss optical transmission using a CMOS-compatible process. Moreover, the Si waveguide has a larger refractive index compared with the LN waveguide, resulting in a smaller bending radius and higher degrees of integration.

In the design of LN modulators, the *x*-cut or *z*-cut LN film is commonly used. To fully exploit the largest EO coefficient *r*_33_, the applied electric field should be paralleled with the optical axis (*z*-axis) of the LN crystal. Although LN has a wide transparent window from 0.25 to 5.3 μm, most of the reported LN MIR modulators operate around the 2–3 μm bands. There are two main reasons. Firstly, the 2-μm spectrum can be used as an alternative option for traditional optical communication bands. Secondly, SiO_2_ cladding or polymer bonding agents would induce high optical losses in the MIR spectrum [60], which hinders the application of LNOI platforms in longer MIR bands.

**Figure 5:**
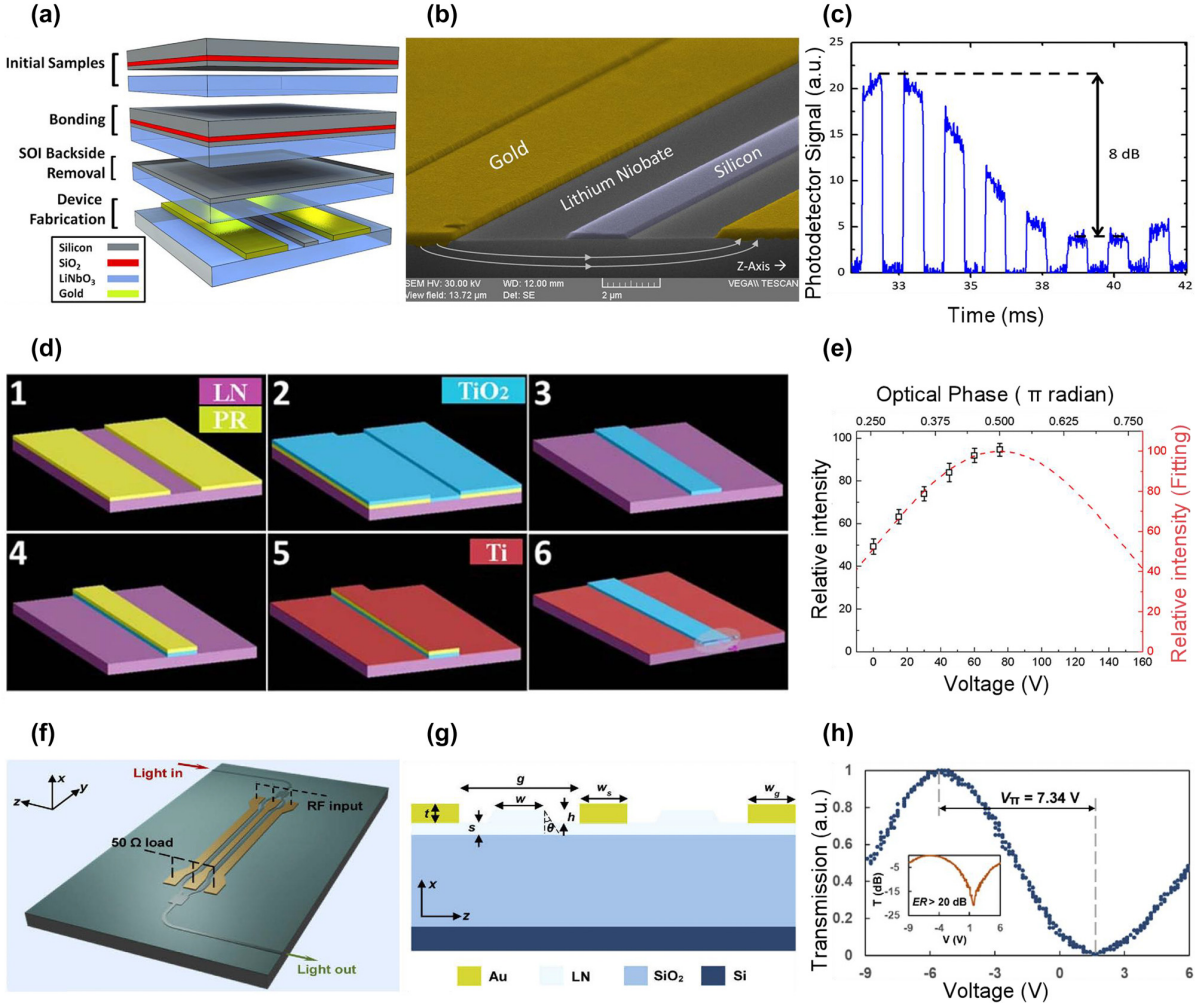
Representative MIR LN modulators. (a) Direct bonding process to form Si-on-LN platform. (b) Scanning electron microscopy image of the MIR LN modulator working at 3.39-μm wavelength. (c) The characteristics of AC extinction ratio under square-wave signal. (d) The lift-off processes of depositing TiO_2_ waveguides on LN substrate. (e) Relative light intensity as a function of applied DC voltage. The red dashed curve is the fitting result. (f) Schematic of MIR LNOI modulator operating at 2-µm wavelength. (g) Cross-section of LN ridge waveguides on Si substrate. (h) Optical transmission under DC voltage, the inset shows the DC extinction ratio. Figures reproduced with permission from (a), (b), (c) Ref. [65], © The Optical Society; (d), (e) Ref. [67] licensed under a Creative Commons Attribution (CC BY) license; (f), (g), (h) Ref. [59], © The Optical Society.

Some of the MIR LN modulators employ rib-loaded configurations where the different waveguide core materials are placed on top of the LN EO active layer to avoid the potential material losses associated with the direct etching of LN films. In 2014, Chiles and Fathpour proposed a MIR LN modulator operating at 3.39 μm [65]. As illustrated in Figure 5(a), they bonded silicon on top of the LN substrate to form Si waveguides. The fabricated MZI-structured modulator [Figure 5(b)] is characterized to have the modulation efficiency of 26 V⋅cm, insertion loss of 3.3 dB, and AC EO of 8 dB, as shown in Figure 5(c). Recently, a MIR LN modulator operating at 3.78 μm has been reported [66]. The modulator utilizes monocrystalline Si waveguides-based devices integrated on LN crystal substrate using the transfer printing method. This modulator achieves a half-wave voltage-length product of 12.3 V⋅cm, DC EO over 25 dB, and a modulation bandwidth exceeding 5 MHz. Notably, compared to the device presented in Ref. [65] on the same material platform, the device performance has a significant improvement.

In 2019, a MIR LN modulator working at 2.5 μm was proposed by Jin et al., using Titanium Dioxide (TiO_2_) as the core guiding material [67]. As illustrated in Figure 5(d), the TiO_2_ waveguide with a wide transparent window up to 8 μm is deposited onto the LN substrate by a lift-off process. The researchers chose to use a *z*-cut LN thin-film in combination with the planar electrodes, therefore the modulator utilizes the EO coefficient *r*_31_ instead of the largest *r*_33_. The measured *V*_π_⋅*L* of this modulator is 50 V⋅cm, with a driven voltage of 150 V [Figure 5(e)]. If *r*_33_ is utilized in *z*-cut LN thin-film, a vertical electrode design is required, which also implies a more complicated fabrication process. In 2021, Pan et al. reported the first MIR MZM using etched LN as the core waveguides [59], the device configuration and cross-section are shown in Figure 5(f) and (g), respectively. The demonstrated modulator exhibits the *V*_π_·*L* of 3.67 V⋅cm [Figure 5(h)] and the on-chip loss of 6 dB, a 25 Gbit/s data rate is achieved in OOK modulation format, which is comparable with the silicon carrier-depletion type MZM working in the same 2-µm band [25].

### 3.2 BTO MIR modulators

The maximum EO coefficient of BTO is 43 times higher than that of LN [68], such a high electro-optic response makes BTO a promising material platform for EO modulators. The qualities of the BTO thin film such as defects, strain, grain size, etc. will influence its linear and non-linear optical properties. If the fabricated thin films are of poor quality (i.e., multi-domain, polycrystalline), then there will be a large deviation between the simulated and experimentally measured values of the modulator. Therefore, the fabrication of the BTO thin film is a critical step. In some reports, BTO film is grown on the SOI platform by molecular beam epitaxy (MBE) with the help of a strontium titanate (STO) seed layer [54] or deposited on other low-refractive-index substrates such as lanthanum aluminate (LAO) [69] and dysprosium scandate (DSO) [70] by KrF excimer pulsed laser. Moreover, a BTO-on-SiO_2_ platform is demonstrated using Al_2_O_3_ as the bonding interface [54].

**Figure 6:**
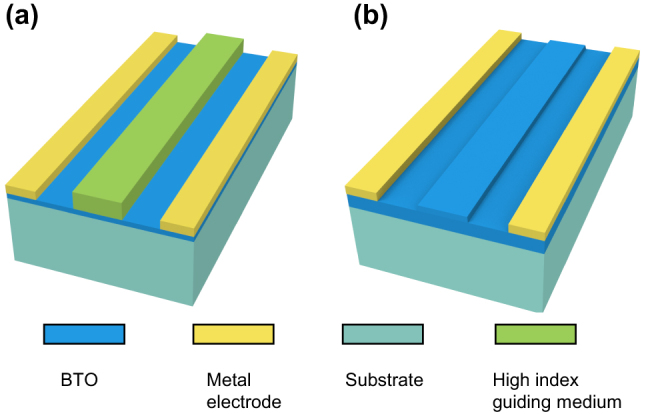
The design of phase shifters for MIR BTO modulators. The design using (a) rib-loaded BTO thin-film, (b) an etched BTO ridge waveguide.

BTO modulators operating in the NIR spectrum exhibit excellent performance due to their ultra-high EO coefficients, such as high modulation speed up to 50 Gbit/s for data communication [54], high modulation efficiency of 0.2 V⋅cm, and low power consumption of 100 nW [71]. BTO material is transparent to light up to 7 μm, to broaden the operating band of BTO modulators to the MIR spectrum, several schemes are proposed, including the use of new waveguide materials such as TiO_2_ [72] and the selection of new substrate materials such as lanthanum aluminate (LAO) [69]. Most of the current designs using BTO as the EO active layer choose not to etch the BTO thin film, but to deposit another high-refractive-index material such as a-Si or titanium dioxide (TiO_2_) acting as the guiding medium, and the BTO layer is sandwiched between the substrate and waveguide, which is shown in Figure 6(a). In this rib-loaded BTO thin-film waveguide configuration, there are some unavoidable problems including: (1) Most of the optical field is confined in the high-index guiding material, instead of BTO active layer, which induces the insufficient optic-electric field overlap and refractive index modulation. (2) If the Si is selected as the guiding medium, such design poses the additional problem of carrier migration in Si waveguides, resulting in an undesired plasma dispersion effect caused by strong electric fields [54]. Therefore, in recent years, researchers etch the BTO layer to form ridge waveguides by using argon-ion milling, inductively coupled plasma (ICP) etching or by wet chemical etching for electro-optic modulation [70, 73], the device configuration is illustrated in Figure 6(b). Moreover, etching methods for BTO layers can be further explored so that some structures such as slot waveguides can be then achieved for better optical confinement and optic-electric overlap [74, 75].

Different from the trigonal structure of the LN crystal, the ferroelectric tetragonal structure of the BTO has a crystallographic point group of *P*4*mm*, explaining the non-zero EO coefficients in the unclamped (zero-stress) bulk crystal are *r*_33_ (∼105 pm/V), *r*_13_ = *r*_23_ (∼10.2 pm/V) and *r*_42_ = *r*_51_ (∼1300 pm/V) [68]. In order to utilize the maximum coefficient *r*_42_ for enhanced EO performance, a well-oriented *c*-axis BTO thin-film is preferred. In 2021, Cao et al. reported a *c*-axis aligned single-crystalline BTO thin film, where the waveguide can be located in any direction and electrodes can be placed in the same plane with the waveguide [70]. Moreover, they etched a BTO ridge waveguide to achieve up to 92 % of the light confinement in the BTO active layer.

Limited studies have been published on MIR modulators using BTO materials. Jin et al. reported a BTO thin-film modulator operating around the 2.6-μm band, with a modulation depth of 8.45 dB and a *V*_π_·*L* of 11 V⋅cm [72]. Although the BTO materials have been approved to have a large EO coefficient, the measured effective electro-optic coefficient of BTO-based optical waveguide in the MIR is only 119.7 pm/V, which is three times smaller than its value in the NIR. It is mainly due to the relatively low optical power fraction of the BTO EO active layer [71]. To further improve the modulation efficiency, bringing it closer to the theoretical limit of 0.42 V⋅cm [72], the key method is to enhance electro-optic overlap in the active layer by optimizing the structural parameters of the waveguides and the gap between electrodes, including: (1) Enhance electric field within the BTO layer by increasing the electrode thickness. (2) Improve mode confinement in BTO layer. (3) Replace the substrate material SiO_2_ with other low index materials to circumvent the large optical absorption in the 2.6–2.9 μm range. In conclusion, BTO is a promising material for MIR modulators, but high-performance BTO modulators need to be further investigated.

### 3.3 Benchmark of ferroelectric oxides MIR modulators

The reported Pockels EO modulators working in the MIR spectrum are summarized in Table 2. Critical parameters (e.g., modulation efficiency, optical response, insertion loss, modulation depth, etc.) that characterize device performance, as well as the material platform and the configuration of the EO active layer are listed. As seen in Table 2, the state-of-the-art MIR LN waveguide-based modulator exhibits a modulation efficiency of 3.67 V⋅cm at 2-μm wavelength [59]. When extending the operating wavelengths to the longer MIR band, the deterioration in the modulation efficiency is observed, which can be attributed to the inadequate electro-optic overlap and optical confinement in the EO active films or waveguide structures. Additionally, for the same reasons, although the measured effective EO coefficient in the MIR BTO modulator is about four times larger than its LN counterpart [76], the driven voltage is as high as 55 V.

**Table 2:** Benchmark of LN and BTO MIR modulators.

Year	Wavelength	Active	*V*_π_·*L* (V⋅cm)	ER (dB)	3 dB bandwidth	Insertion loss	Vg (V)	Structure &	Ref.
	(µm)	material			(GHz)	(dB)		platform	
2021	2	LN	3.67	>20 DC	>22	6	6	LN waveguide LN-on-Insulator platform	[59]
2019	2.5	LN	50	NA.	NA.	NA.	150	LN thin film TiO_2_-on-LN Platform	[67]
2022	3	LN	12	40.7 DC	12 (Theoretical)	0.45	24	LN waveguide LN-on-Insulator platform	[76]
2014	3.39	LN	26	8 AC	0.000023	3.3	∼70	LN substrate Si-on-LN platform	[65]
2022	3.78	LN	12.3	25.2 DC	>5 MHz	NA.	20.8	LN substrate Si-on-LN platform	[66]
2020	2.6	BTO	11	8.45 DC	NA.	NA.	55	BTO thin film BTO-TiO_2_-Insulator platform	[72]

Furthermore, the widespread applications of LN and BTO Pockels crystals in the MIR spectrum are constrained by the immaturity of the manufacturing processes and the limited choice of substrates and guiding materials. Consequently, besides further development of the fabrication and integration processes of the two EO crystals mentioned above, exploring other EO crystal materials that are easier to fabricate using CMOS processes may be an alternatively feasible solution.

## 4 Two-dimensional material-based MIR modulators

In recent years, the rapid development of 2D materials (e.g., graphene [77–79], black phosphorus (BP) [80–82], and transition metal chalcogenides (TMDs) [83–85]) has broken the restrictions on properties of traditional materials and provided opportunities to explore new principles and configurations for functional photonic devices. By exploiting the properties of high carrier mobility and broadband light absorption of 2D materials, high-speed, broadband [86, 87], and low-insertion-loss [88] modulators have been proposed and demonstrated.

In this section, the working principles and properties of photonic waveguide-based graphene modulators and plasmonic waveguide-based graphene modulators are presented. Another emerging 2D electro-optic (EO) material BP is also mentioned, which shows promising perspectives for MIR modulators. In addition, some representative modulators in terms of modulation methods and device performance are briefly introduced.

### 4.1 Graphene MIR modulators

Since Novoselov et al. first fabricated stable monolayer graphene films by mechanical exfoliation in 2004, graphene has opened new prospects as an attractive candidate for optoelectronic devices in integrated photonics due to its remarkable optical properties and compatibility with CMOS processing [89]. The thickness of single-layer graphene is about 0.34 nm [90]. Unlike metals with plenty of free charges, graphene is a semi-metal, in which the conduction and valence bands converge at the Dirac point. This special zero-gap band structure allows its Fermi level to vary significantly with carrier concentration near the Dirac point [91]. Electro-absorption modulation in graphene is accomplished by artificially tuning the Fermi level (*E*_*F*_) through chemical doping [92] or bias voltage [91] that can change the carrier concentration. When the incident photon energy exceeds 2*E*_*F*_, the graphene absorbs the light. Conversely, when the incident photon energy is less than 2*E*_*F*_, the interband transition is prohibited due to the Pauli blocking, resulting in low absorption [93]. When graphene operates in the Pauli blocking regime, it enables phase modulation for complex modulation formats, since the change of Fermi level caused by intraband transition can also affect the real part of the refractive index of graphene [94].

**Figure 7:**
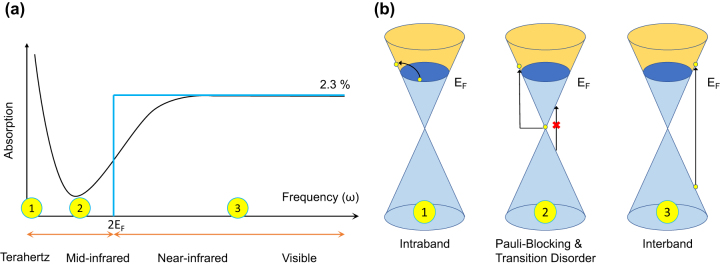
Schematic of broadband absorption of p-doped graphene with specific E_F_ at different optical frequencies (*ω*). (a) Illustration of graphene absorption spectra under various frequencies [93, 96, 106]. A Drude peak can be observed at the terahertz band; the minimum absorption occurs in the MIR band owing to Pauli blocking and transition disorder; a universal absorption of up to 2.3 % is obtained from NIR to visible band. (b) Energy diagram of p-doped graphene. Depending on the various incident photon energy compared to a fixed value of 2 E_F_, interband transition, intraband transition, and Pauli-blocking happens.

For better understanding, Figure 7(a) and (b) illustrate a broadband absorption by p-doped graphene with specific *E*_*F*_ under different frequencies (*ω*) of light. Graphene interacts strongly with electromagnetic waves ranging from visible to NIR region, absorbing up to 2.3 % of light [95]. In this wavelength range, interband transitions dominate as a result of relatively large photon energy. A Drude peak response can be observed in the terahertz band, where the intraband carrier’s absorption is more significant due to lower photon energy than thermal energy [96]. In the MIR band, when photon energy is around 2*E*_*F*_, there is a momentum disorder in photon transition due to the switch from interband to intraband transition [93, 97], so that the light absorption is relatively reduced.

Graphene can be coupled with free-space light or evanescent waves in waveguides to realize amplitude or phase modulation. Several photonic waveguide-based graphene modulators operating in the NIR band have been reported with excellent performance [93, 97–101]. However, research on the MIR band is still at the exploratory stage. Since the confinement of the optical modes by monolayer graphene attenuates with increasing wavelength, the modulation depth (MD) consequently decreases [102]. Plasmonic graphene modulators with effective mode areas several orders of magnitude smaller than photonic modes are proposed to provide extreme light confinement so as to improve modulation efficiency [103].

#### 4.1.1 Photonic waveguide-based graphene modulators

When designing a waveguide-based graphene modulator, the first consideration should be the selection of the guiding medium and gate dielectric material. Both materials should be transparent to a certain MIR band and capable to be integrated with the active layer of 2D materials. The standard configuration of the photonic waveguide-based graphene modulator is a monolayer graphene-insulator-waveguide medium capacitor [98]. To further increase the modulation efficiency, a double layer of graphene can be used [87].

**Figure 8:**
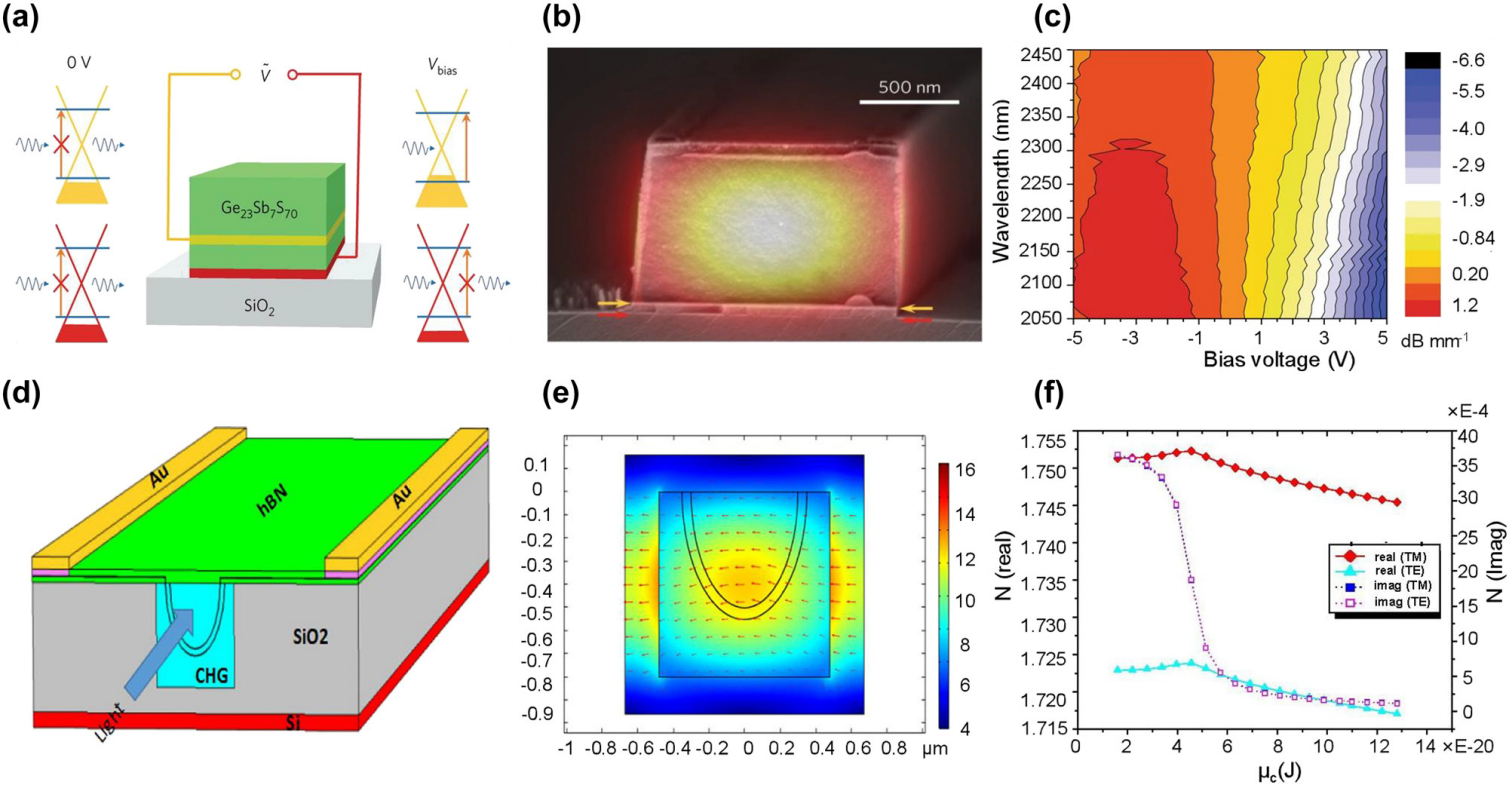
Representative MIR waveguide-based graphene modulators. (a) Schematic of the double-layer graphene modulator using Ge_23_Sb_7_S_70_ as guiding medium. (b) SEM image of the cross-section of the waveguide. The red and yellow arrows indicate the position of the two graphene layers, respectively. (c) The measured modulation depth under various wavelengths and applied voltages. (d) Schematic of the double-layer polarization-independent graphene modulator using Ge_23_Sb_7_S_70_ as guiding medium. (e) The electric field profile for TM mode light. (f) Illustration of the refractive index (real part and imaginary part) as the function of chemical potential. Figures reproduced with permission from: (a), (b) (c) Ref. [104], Springer Nature; (d), (e), (f) Ref. [107], licensed under Creative Commons Attribution 4.0 International License.

In 2017, Lin et al. demonstrated the first waveguide-based MIR graphene modulator with other photonic functional devices including optical switches, polarizers, and photodetectors, providing a solution for the integration of on-chip photonic devices using 2D materials [104]. The configuration of the waveguide modulator is shown in Figure 8(a) and (b). Double-layer graphene is used to increase the relative weak absorption of light by graphene in the MIR band. Ge_23_Sb_7_S_70_, one of chalcogenide glasses (ChGs), is used as waveguide material, gate dielectric, and passive barrier of the graphene sheet. ChG is transparent in the MIR band (from 2 to 10 μm [102]), which is suitable for integration with 2D materials due to its high deposition rate at room temperature [105] and good van der Waals adhesion with various substrates. The 700-μm-long waveguide is designed as an amplitude modulator and operates in the 2.05–2.45 μm band, achieving an MD of 8 dB/mm [Figure 8(c)] and a 3-dB bandwidth of 23 kHz. Further optimization is necessary to increase the modulation speed and modulation efficiency.

In 2021, the same group reported a concept of a polarization-independent MIR graphene modulator using the Ge_23_Sb_7_S_70_ waveguide as well [107]. The bilayer graphene is bent into a U-shape and embedded in a Ge_23_Sb_7_S_70_ insulating layer as depicted in Figure 8(d) and (e). By carefully designing the bending radius of the two graphene layers, the device can support both TE and TM mode light and overcome its dependence on polarization [Figure 8(f)]. The 200-μm-long device operates at 2.2 μm, with 16 dB MD, <1 dB insertion loss, and 136 GHz estimated modulation bandwidth. However, the viability and film quality of the bending graphene layers needs to be assessed and the performance of the fabricated devices needs to be further verified.

To our best knowledge, all waveguide-based graphene modulators in the MIR region have been reported as electro-absorption modulators. It is necessary to investigate graphene phase modulators for advanced modulation format to improve the spectral efficiency of the single transmission channel.

#### 4.1.2 Plasmonic waveguide-based graphene modulators

In order to enhance the light-graphene interaction in the MIR band, Wang et al. demonstrated subwavelength graphene micro-ribbon arrays by oxygen plasma etching and excited the plasmon resonance successfully [108]. Surface plasmon polariton (SPP) is an electromagnetic wave that propagates on the interfaces between two media with opposite signs of the dielectric constant and decays exponentially along the direction away from the interface. Graphene has a negative dielectric constant [93], which perfectly compensates for the weak confinement of metal SPP in the MIR band.

The plasmonic graphene modulators have been widely studied for amplitude or phase modulation of spatial light. In addition to the use of single-layer graphene or lithographically defined graphene slabs or ribbons for coupling with light transmitted in the free space [109–113], the integration of graphene with metamaterials and metallic nanostructures, such as plasmonic nanoantenna arrays [114–117] and Fano resonant plasmonic nanostructures [118, 119], enables the devices with increased tunability and better performance (i.e., large MD and high modulation speed).

The plasmonic waveguide-based graphene modulator functions on a similar principle to the conventional photonic waveguide-based graphene modulator mentioned above, which is to modulate the Fermi level of graphene by applying gate voltage actively. However, in the plasmonic modulator, atomically thin graphene acts as a 2D surface plasmon polariton (SPP) waveguide [109], where light is transmitted within the 2D space of the graphene surface, making it more sensitive to the carrier concentration change of the graphene.

**Figure 9:**
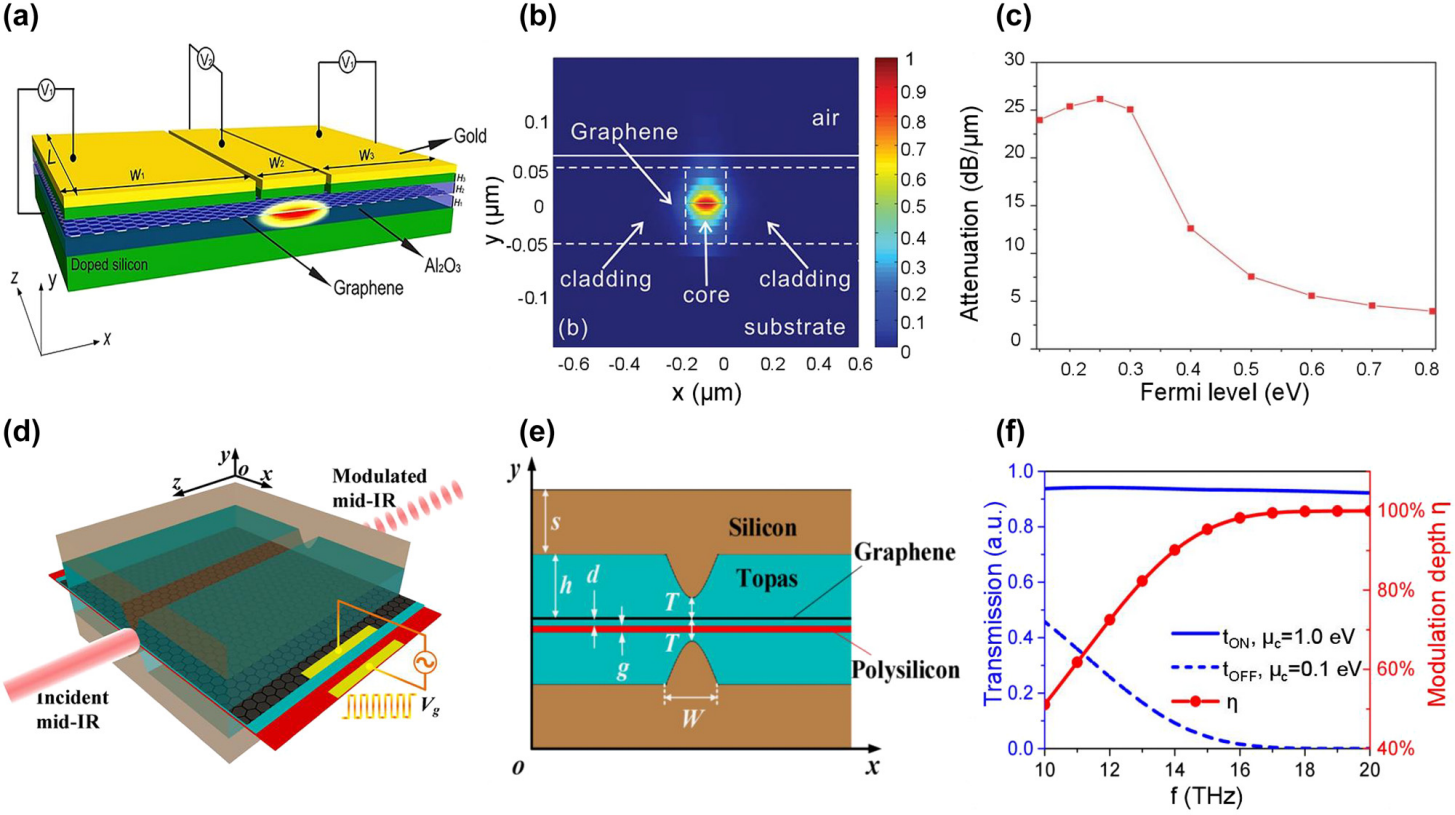
Representative MIR plasmonic graphene modulators. (a) Schematic of the plasmonic graphene modulator. (b) The electric field distribution of the 2D plasmonic waveguide at 40 THz frequency. (c) The light attenuation versus Fermi level of the cladding at the frequency of 37 THz. (d) Schematic of the plasmonic graphene modulator. (e) Cross-section of the plasmonic waveguide. (f) The ON/OFF state light transmission (left) and corresponding modulation depth (right) as functions of frequency. Figures reproduced with permission from: (a), (b) (c) Ref. [109], John Wiley and Sons; (d), (e), (f) Ref. [120], © The Optical Society.

A plasmonic modulator was numerically investigated by J Lao et al. [109]. As shown in Figure 9(a) and (b), monolayer graphene is sandwiched between two dielectric mediums (aluminum oxide) to form cross interfaces with opposite values of dielectric constants. Since the effective index of the SPP waveguide is inversely proportional to the complex surface conductivity of the graphene, by applying different voltages to the waveguide cladding, the Fermi level of the graphene is adjusted and the effective index of the cladding is modified. When the Fermi level of the graphene at the cladding is lower than that of the core, the high attenuation mode of the SPP waves transmitted on the graphene surface is excited, and most of the light is absorbed by the cladding. As shown in Figure 9(c), an MD of 21.5 dB/μm can be achieved at a working frequency of 37 THz (∼8.1 μm) by adjusting the Fermi level of the overlay graphene from 0.8 eV to 0.2 eV. In 2018, a hybrid plasmonic modulator was reported with a broadband modulation range from 10 to 20 THz (∼15–30 μm) [120]. As shown in Figure 9(f), an ultra-high MD approaching 100 % is obtained at light frequencies higher than 18 THz (∼16.7 μm). Such excellent modulation properties benefit from its unique hybrid parabolic-ridge waveguide structure presented in Figure 9(d) and (e), which enhances the confinement of the fundamental SPP mode on the graphene layer. Moreover, in 2023, a Fabry–Perot (FP)-based tunable modulator was proposed [101]. This modulator benefited from surface plasmon resonances supported by graphene-coated gratings, allowing for a very high modulation depth of nearly 100 % across a wide frequency range from THz (nearly 1 THz) to MIR (90 THz). It is worth noting that research on MIR plasmonic waveguide-based graphene modulators is still in a relatively preliminary stage, constrained by their complex structures and immature integration process with 2D materials. To the best of our knowledge, no characterization results of fabricated modulators have been reported in this field.

### 4.2 Black phosphorus (BP) modulators

Black phosphorus (BP) is another attractive 2D material that has been extensively investigated for optoelectronics applications in the MIR range owing to its excellent properties, including high carrier mobility (over 1000 cm^2^ V^−1^ s^−1^ in few-layer BP), thickness-dependent bandgap (2 eV–0.3 eV for monolayer and bulk BP, respectively.), and strong interactions with light [120–123]. Various optoelectronic functional devices have been experimentally demonstrated, including photodetectors [20, 124, 125], modulators [126–128], and light emission devices [129–131]. Nevertheless, 2D BP is chemically unstable, ambient degradation of the material imposes additional challenges on the integration process [132]. Once large-scale fabrication of high-quality BP thin films can be realized, photonic devices using BP material will have great application prospects.

**Figure 10:**
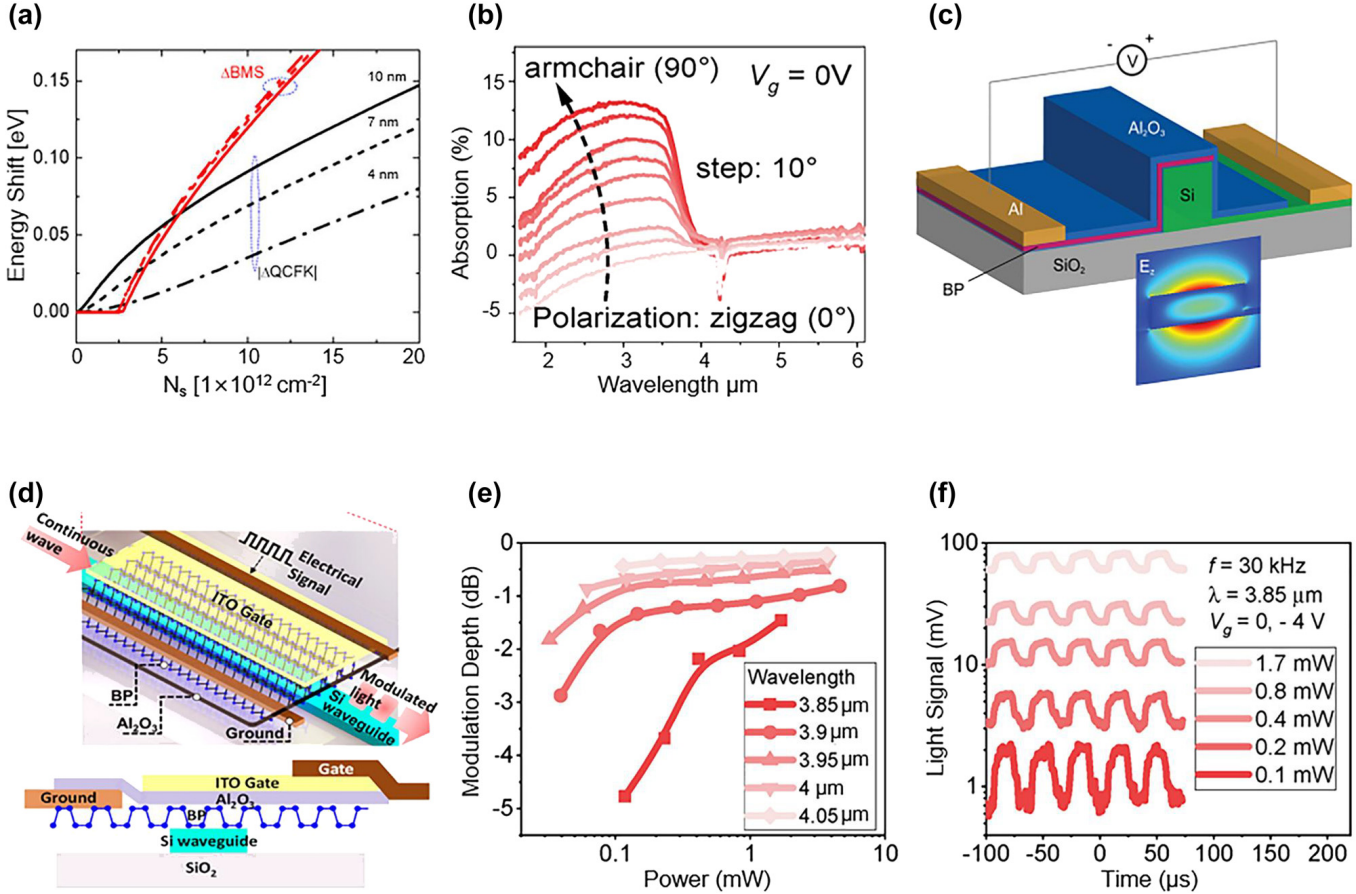
Representative MIR BP modulators. (a) Bandgap shift as a function of carrier concentration with various BP thicknesses from 4 to 10 nm. (b) Light absorption of BP versus wavelength. (c) Schematic of the BP electro-absorption modulator. (d) Schematic and cross-section of the BP electro-absorption modulator. (e) Modulation depth as a function of light power. (f) Modulated light signal under various light power at 3.85-μm wavelength. Figures reproduced with permission from: (a), (c) Ref. [134], American Chemical Society; (b), (d), (e), (f) Ref. [135], Elsevier.

The EO response of BP flake arises from the combination of the quantum confined Franz–Keldysh (QCFK) effect and the Burstein–Moss (BM) effect [133]. The QCFK effect is the direct change in the energy bandgap triggered by the external voltage. While the BM effect is the change of effective bandgap due to the modulation of the Fermi energy level by an applied electric field, where the absorption edge becomes less sharp than the QCFK effect [21]. Both of the effects modulate the light by tuning the absorption edge. As shown in Figure 10(a), at lower carrier concentration, the QCFK effect is dominant, whereas the BM effect is dominant at higher carrier concentration [134]. In addition, BP exhibits an anisotropic electro-optic response [128]. As depicted in Figure 10(b), the optical conductivity along the armchair direction (crystalline axis along the *x*-axis) is higher than that of the zigzag direction (crystalline axis along the *y*-axis), implying that the carrier concentration is higher in the direction of the armchair. If the BM effect is the dominant modulation mechanism, the light needs to be polarized in the armchair orientation, where the absorption by BP is much stronger than that along the zigzag direction [135]. Therefore, when designing a BP modulator, it is necessary to consider the mode of transmitted light, the relative position between the crystal phase of the BP film (armchair direction) and waveguides, as well as the layout of the electrodes.

A waveguide-based BP modulator is proposed by Charles Lin et al. for conceptual verification [134]. As illustrated in Figure 10(c), the structure of the BP modulator consists of the Si waveguide, which is covered by BP thin film, with an Al_2_O_3_ layer acting as a spacer and passivation layer. By increasing the thickness of BP from 6 nm to 20 nm, the bandgap of BP is reduced, and a transition from BM-dominated EO response to QCFK-dominated EO response can be observed. When the BP modulator operates in the QCFK regime, the multilayer BP modulator shows stronger light absorption and lower power consumption compared to the graphene modulator using the same configuration. The calculation indicates that in BP thin-films, the QCFK effect is superior to the BM effect for EO response.

In 2021, Huang et al. demonstrated a BP modulator with an integrated BP photodetector on the same chip, providing an attainable solution for a compact BP optoelectronic system in the MIR band [135]. Leveraging the waveguide-based design, a 20-nm heavily p-doped BP thin film is transferred onto the Si waveguide as the EO active layer [Figure 10(d)]. The device shows a power consumption of 2.6 pJ within a 225-μm interaction length. The modulation mechanism of the BP modulator is dominated by the BM effect rather than QCFK, as the applied gate voltage is too small to induce the direct change of the bandgap. As shown in Figure 10(e) and (f), the BP modulator exhibits ∼5 dB MD of 120 μW light under −4 V gate voltage. In addition, the MD can be enhanced by further reducing the illumination power, which implies the device is suitable for processing weak light signals.

### 4.3 Benchmark of 2D material-based MIR modulators

The benchmarking of 2D material modulators covering critical device performance (e.g., modulation depth, gate voltage, and insertion loss) and configurations are presented in Table 3. Besides the photonic waveguide-based graphene modulators and plasmonic waveguide-based graphene modulators, MIR BP modulators are included as well.

**Table 3:** Benchmark of waveguide-based graphene and BP MIR modulators.

Year	Active	Remarks	Wavelength	MD (dB/μm)	Insertion loss	Vg (V)	Ref.
	material		(µm)		(dB)		
2017	Graphene^a^	Photonic	2.05–2.45	0.008	NA.	−5–5	[104]
		Double-layer				(OFF-ON voltage)	
2021	Graphene	Photonic	2–2.4	0.08	1	NA.	[107]
		Double-layer					
2022	Graphene	Hybrid plasmonic	8–12.4	74.5 @8 µm	NA.	0–64	[100]
				93 @12.4 µm		(OFF-ON voltage)	
2018	Graphene	Hybrid plasmonic	∼15–16.7	136.6	NA.	0.38–31.33 (OFF-ON voltage)	[120]
2021	Graphene	Hybrid plasmonic	∼16.7–37	2.5	NA.	NA.	[137]
2021	Graphene	Hybrid plasmonic	2.23	0.129	0.038	0.98–3.94 (OFF-ON voltage)	[136]
2017	Graphene	Hybrid plasmonic	8.014	>4	<2	NA.	[138]
2014	Graphene	Hybrid plasmonic	6–8.6	21.5	NA.	NA.	[109]
2023	Graphene	Hybrid plasmonic	3.33–299.792	∼100 %	0.6	<1	[101]
2021	BP^a^	20 nm BP	3.85–4.1	0.033	NA.	−4	[135]
2016	BP	6–20 nm BP	2.1–3.3	NA.	NA.	NA.	[134]

(Works with ^a^ are experimental results, others are simulation results.)

As seen in Table 3, the reported MIR graphene modulators are able to operate at wavelengths ranging from 2 μm to 16.7 μm, benefiting from the broadband absorption of the graphene. Furthermore, compared to the photonic waveguide-based graphene modulator [104], the plasmonic graphene modulator [136] shows significant improvements including modulation depth (5.6 dB vs. 12.9 dB), device length (700 µm vs. 100 µm), and OFF/ON gate voltage (−5–5 V vs. 0.98–3.94 V). As the light–matter interaction has been greatly enhanced by confining SPP waves within the monolayer graphene which is beyond the diffraction limits. Nevertheless, the listed plasmonic modulators only have simulation results as a proof-of-concept, the experimental verification remains a challenge.

Few studies have been reported on MIR BP modulators. In Ref [135], the BP modulators can only modulate the light with low illumination power, showing a modulation depth of 5 dB. To further improve the device performance, some resonant structures such as BP/other 2D material QWs might be a feasible option.

In addition, further studies on the large-scale fabrication processes and improvements in the stability of 2D materials are demanded to take advantages of their superior optical properties in practical applications for integrated photonics.

## 5 Summary and future outlook

In conclusion, we reviewed most of the recent research advances in MIR modulators utilizing various EO active materials. Each type of modulator possesses distinct advantages that can be applied to diverse application scenarios.

Firstly, Si and Ge electro-refractive modulators using the plasma dispersion effect exhibit impressive performance in the wavelength range of 2–4 μm. A carrier-depletion Si modulator working at 2-μm wavelength shows the modulation efficiency of 1.6 V⋅cm and a record data rate up to 80 Gbit/s, paving the way for the development of the optical communication at the 2-μm band [26]. For the wavelength above 4 μm, most of the reported Si and Ge modulators are based on electro-absorption. Since the free carrier absorption dramatically increases with wavelength, it is more suitable to be the dominant modulation mechanism. The maximum operating wavelength is currently at 10.7 µm [29]. Albeit these EAMs cannot perform phase modulation and advanced modulation formats for high-speed data transmission, they can be used for MIR spectral sensing which requires operating speeds of 10 Hz–100 kHz [139].

Secondly, Pockels effect-based LN and BTO MIR modulators have been investigated to work at 2–3 μm band. A MIR LN modulator with a modulation efficiency of up to 3.67 V⋅cm and an electrical bandwidth of 22 GHz was reported in 2021 [59]. Although the effective EO coefficient in the MIR BTO thin-film modulator is approximately four times larger than its LN counterpart, the driven voltage is as high as 55 V due to the relatively low modulation efficiency of 11 V⋅cm, which is caused by the inadequate electro-optic overlap and optical confinement.

Finally, graphene and BP MIR electro-absorption modulators have wide operating wavelength ranges and ultra-compact footprints. It has been experimentally demonstrated that the MIR graphene [104] and BP [135] modulators with modulation lengths of only a few hundred micrometers can achieve modulation depth of 8 dB/mm and 33.3 dB/mm, respectively. However, the 3-dB bandwidth is relatively small (23 kHz for graphene modulator and 400 kHz for BP modulator). As long as the contact resistance is reduced by optimizing the device configuration and the challenges of large-scale fabrication are overcome, 2D material-based modulators will show outstanding performance such as compact size, ultra-high modulation depth and modulation speed for practical applications.

When extending the operating wavelength of EO modulators from the NIR band to the MIR band, we could observe an obvious performance degradation for most of the EO modulators, except for plasma-dispersion-based electro-absorption modulators. Although progresses have been made, according to the authors’ understanding, the research of MIR modulator is still in the infancy stage. There are a lot of interesting research areas in integrated MIR modulators that are worthy to pay attention to, including:

1. Developing high-speed Si electro-refractive modulators working at 2–4 μm band. Although Si modulators for the 2-μm band have been extensively studied and show convincing performance, only one Si modulator operating at the 3.8-μm band has been reported [22]. The high absorption of the SiO_2_ substrate in the range of 2.6–2.9 μm and above 3.6 μm limits the extension of the applicable spectrum of the SOI platform. Thus, the study of the Si modulators using other platforms, such as the silicon-on-porous silicon [140], silicon-on-sapphire [141, 142], silicon-on-calcium fluoride [143], and silicon-on-silicon nitride [144] could be a promising research direction.
2. Exploring Ge-based modulators for improved performance over a wide wavelength range. In the shorter MIR band (<4 μm), the carrier-depletion-based Ge electro-refractive modulators could be studied to obtain broader RC bandwidth, faster modulation speed, and lower power consumption. In addition, at the wavelengths above 4 μm, further studies on Ge-based electro-absorption modulators with improved performance may open up new prospects for spectroscopic sensing.
3. Exploring MIR EAMs utilizing QCSE. QCSE has been widely employed in high-performance quantum well modulators, offering attractive features such as sub-micrometer modulation lengths, sub-picosecond operating speeds, and large modulation bandwidths. Numerous publications have recently explored NIR modulators utilizing QCSE, both with III–V [145–147] and group-IV [148, 149] based QWs. Effective coupling between multi-quantum-well structures and passive waveguides can be achieved through adiabatic taper couplers or resonant taper couplers. However, only III–V spatial light EAMs with quantum well structures have been reported to date [150, 151], the current literature lacks reports on waveguide-based MIR III–V modulators. In addition, research on GeSn/SiGeSn EAMs operating beyond the 2-μm wavelength range remains limited. Further investigation into MIR EAMs utilizing QCSE holds promising potential for achieving high-performance modulation outcomes.
4. Extending the working wavelength of the Pockels-based LN and BTO modulators to longer MIR bands. Since the reported LN and BTO MIR modulators operate in 2–3 μm band, LN modulators operating in 3–5 μm band and BTO modulators operating in 3–7 μm band are worth being investigated. Moreover, in order to take full advantages of BTO materials in the MIR band, modulators using etched BTO waveguides on SiO_2_ insulators could be exploited in wavelengths less than 4 μm. In a longer wavelength range, BTO on other low-refractive-index substrates such as LAO and DSO could be explored to demonstrate high performance MIR modulators.
5. Experimentally demonstrating graphene waveguide-based phase modulators and plasmonic modulators operating in the MIR band. In NIR band, the reported graphene phase modulators in the form of a graphene–insulator–silicon (GIS) or a graphene–insulator–graphene (GIG) capacitor exhibit convincing modulation efficiency, due to the strong electro-refractive effect when operating in the Pauli blocking regime. It is also necessary to explore MIR graphene phase modulators with advanced modulation formats. Additionally, although simulation results show excellent device performance of MIR graphene plasmonic modulators, there lacks experimental verification.
6. Integrating MIR modulators with other functional components to form MIR PICs. By investigating the suitable photonic platforms to support versatile functions, we could expect compact, high-performance on-chip systems which can be used in various applications including trace gas detection, medical imaging, and environmental monitoring.
